# Supplementary material for: Isolation and identification of an isoflavone reducing bacterium from feces from a pregnant horse
Source: PLoS One. 2019 Nov 18;14(11):e0223503. doi: 10.1371/journal.pone.0223503 (PMC6860936; doi:10.1371/journal.pone.0223503)
Supplement: S1 Table — (DOCX) [file pone.0223503.s001.docx]

| Treatment | | Inoculated strain HXBM408 | | | | Uninoculated strain HXBM408 | | | |
| --- | --- | --- | --- | --- | --- | --- | --- | --- | --- |
|  |  | 1 | 2 | 3 | Average value | 1 | 2 | 3 | Average value |
| 0 h | DAI | 20.2 | 20.16 | 20.22 | 20.19 | 20.13 | 20.09 | 20.11 | 20.11 |
|  | DHD | N/D | N/D | N/D | N/D | N/D | N/D | N/D | N/D |
|  | Equol | N/D | N/D | N/D | N/D | N/D | N/D | N/D | N/D |
| 48 h | DAI | 18.84 | 18.87 | 18.87 | 18.86 | 20.11 | 20.07 | 20.09 | 20.09±0.02 |
|  | DHD | 0.12 | 0.11 | 0.11 | 0.11 | N/D | N/D | N/D | N/D |
|  | Equol | N/D | N/D | N/D | N/D | N/D | N/D | N/D | N/D |

S1 Table. Concentration of daidzein and its metabolites in culture medium before and after culture of HXBM408
