# Supplementary material for: Isolation and identification of an isoflavone reducing bacterium from feces from a pregnant horse
Source: PLoS One. 2019 Nov 18;14(11):e0223503. doi: 10.1371/journal.pone.0223503 (PMC6860936; doi:10.1371/journal.pone.0223503)
Supplement: S2 Table — (DOCX) [file pone.0223503.s002.docx]

| A600 | | | | | pH | | | |
| --- | --- | --- | --- | --- | --- | --- | --- | --- |
| Time | 1 | 2 | 3 | Average value | 1 | 2 | 3 | Average value |
| 0h | 0.0025 | 0.0044 | 0.0031 | 0.0033 | 7.12 | 7.20 | 7.09 | 7.14 |
| 1h | 0.0433 | 0.0529 | 0.0427 | 0.0463 | 7.14 | 7.08 | 7.08 | 7.10 |
| 2h | 0.0967 | 0.1014 | 0.0982 | 0.0988 | 6.98 | 7.04 | 6.96 | 6.99 |
| 4h | 0.6464 | 0.6921 | 0.6626 | 0.6670 | 6.82 | 6.81 | 6.84 | 6.82 |
| 6h | 0.8991 | 0.9002 | 0.8621 | 0.8871 | 6.79 | 6.72 | 6.81 | 6.77 |
| 8h | 0.9610 | 0.9911 | 0.9887 | 0.9803 | 6.82 | 6.78 | 6.86 | 6.82 |
| 10h | 1.0021 | 1.0151 | 1.0210 | 1.0127 | 6.88 | 6.79 | 6.84 | 6.84 |
| 14h | 1.0303 | 1.0256 | 1.0274 | 1.0278 | 6.82 | 6.84 | 6.85 | 6.84 |
| 18h | 1.0469 | 1.0338 | 1.0307 | 1.0371 | 6.88 | 6.82 | 6.85 | 6.85 |
| 22h | 1.0146 | 1.0303 | 1.0312 | 1.0254 | 6.87 | 6.86 | 6.92 | 6.88 |
| 26h | 1.0582 | 1.0124 | 1.0363 | 1.0356 | 6.88 | 6.87 | 6.86 | 6.87 |
| 48h | 1.0014 | 1.0089 | 1.0315 | 1.0139 | 6.90 | 6.88 | 6.90 | 6.89 |

S2 Table. Growth curve and pH change
