# Supplementary material for: Isolation and identification of an isoflavone reducing bacterium from feces from a pregnant horse
Source: PLoS One. 2019 Nov 18;14(11):e0223503. doi: 10.1371/journal.pone.0223503 (PMC6860936; doi:10.1371/journal.pone.0223503)
Supplement: S3 Table — (DOCX) [file pone.0223503.s003.docx]

| Temperature（℃） | 1 | 2 | 3 | Average value |
| --- | --- | --- | --- | --- |
| 22 | 0.0617 | 0.0794 | 0.0772 | 0.0728 |
| 27 | 0.6158 | 0.6019 | 0.5727 | 0.5968 |
| 32 | 0.7934 | 0.7998 | 0.7641 | 0.7858 |
| 37 | 0.9102 | 0.9195 | 0.9114 | 0.9137 |
| 42 | 0.7252 | 0.7148 | 0.7491 | 0.7297 |
| 47 | 0.6661 | 0.6709 | 0.6745 | 0.6705 |
| 52 | 0.0348 | 0.0258 | 0.0334 | 0.0313 |

S3 Table. Effects of Temperature on the Growth of the Strain
