# Supplementary material for: Isolation and identification of an isoflavone reducing bacterium from feces from a pregnant horse
Source: PLoS One. 2019 Nov 18;14(11):e0223503. doi: 10.1371/journal.pone.0223503 (PMC6860936; doi:10.1371/journal.pone.0223503)
Supplement: Attachment — Submitted filename: Comment.docx [file pone.0223503.s004.docx]

Reviewer comment:

1.

Introduction: Tihydodaidzein (THD) maybe corrected in 1^st^ paragraph

2^nd^ line of second para have no reference.

2. Methodology:

Para 1.6, the reference of Decroos K et al, 2005 is not in the reference

3. Result:

Para 2.3, Table 1 written but should be Table 2

4. Reference:

Not arrange alphabetically

Final comment:

The article is written nicely but has some minor mistakes. Overall the article lacks novelty and may not be consider for publication.
