# Supplementary material for: Isolation and identification of an isoflavone reducing bacterium from feces from a pregnant horse
Source: PLoS One. 2019 Nov 18;14(11):e0223503. doi: 10.1371/journal.pone.0223503 (PMC6860936; doi:10.1371/journal.pone.0223503)
Supplement: Attachment — Submitted filename: Response to Reviewers.doc [file pone.0223503.s005.doc]

Reply to academic editor：

1.Introduction: Tihydodaidzein (THD) maybe corrected in 1^st^ paragraph 2^nd^ line of second para have no reference.

Answer: Modifications have been made. The following is the first sentence of the article.

（Dihydro daidzein (DHD) is a metabolite formed by daidzein in the animal intestines through the specific microbial group action. It has higher biological activity than its predecessor of the daidzein, mainly in estrogen-like form. such as estrogen-like effect, antioxidant and enzyme inhibition effect, etc.）

2. Methodology:

Para 1.6, the reference of Decroos K et al, 2005 is not in the reference

Answer: References have been added.

Decroos K, Vanhemmens S, Cattoir S, et al. Isolation and characterisation of an equol-producing mixed microbial culture from a human faecal sample and its activity under gastrointestinal conditions[J]. Archives of Microbiology, 2005, 183(1): 45-55.

3. Result:

Para 2.3, Table 1 written but should be Table 2

Answer: The table number has been modified.

4. Reference:

Not arrange alphabetically

Answer: The literature reference order has been modified.

Additional Editor Comments:

The anaerobic tube was placed in the incubator for 48 hours at 37°C, before samples were taken for HPLC analysis

Incubation temperature at 37°C : Is not standardized anywhere ( Give the reference)

Some new references inside the text are to be cited as the manuscript has references before 2010/2011.

Answer: This method is referred to in the literature such as Decroos K et al.

Decroos K, Vanhemmens S, Cattoir S, et al. Isolation and characterisation of an equol-producing mixed microbial culture from a human faecal sample and its activity under gastrointestinal conditions[J]. Archives of Microbiology, 2005, 183(1): 45-55.

Reply reviewer：

1. Is the manuscript technically sound, and do the data support the conclusions?

The manuscript must describe a technically sound piece of scientific research with data that supports the conclusions. Experiments must have been conducted rigorously, with appropriate controls, replication, and sample sizes. The conclusions must be drawn appropriately based on the data presented.

Reviewer #1: Yes

Reviewer #2: Yes

2. Has the statistical analysis been performed appropriately and rigorously? 

Reviewer #1: I Don't Know

Reviewer #2: No

Answer: The table data has more than three repeated tests, and the standard deviation is added.

3. Have the authors made all data underlying the findings in their manuscript fully available?

The PLOS Data policy requires authors to make all data underlying the findings described in their manuscript fully available without restriction, with rare exception (please refer to the Data Availability Statement in the manuscript PDF file). The data should be provided as part of the manuscript or its supporting information, or deposited to a public repository. For example, in addition to summary statistics, the data points behind means, medians and variance measures should be available. If there are restrictions on publicly sharing data—e.g. participant privacy or use of data from a third party—those must be specified.

Reviewer #1: Yes

Reviewer #2: Yes

4. Is the manuscript presented in an intelligible fashion and written in standard English?

PLOS ONE does not copyedit accepted manuscripts, so the language in submitted articles must be clear, correct, and unambiguous. Any typographical or grammatical errors should be corrected at revision, so please note any specific errors here.

Reviewer #1: Yes

Reviewer #2: Yes

5. Review Comments to the Author

Please use the space provided to explain your answers to the questions above. You may also include additional comments for the author, including concerns about dual publication, research ethics, or publication ethics. (Please upload your review as an attachment if it exceeds 20,000 characters)

Reviewer #1: The manuscript provides imprecise insights in comparison and correlation between faeces of different horse varieties.

Comparison with aerobic bacterial strains is also missing.

Clear evidence with respect to standardization of cultural parameters is not available.

Reviewer #2: The article is written nicely baring few mistakes but discussion part could be improved. The article in my view lacks novelty.

Answer: The purpose of this study was to screen for strains that degrade daidzein from fresh horse manure, rather than for comparisons between different breeds of horses. Moreover, in the past, the screening of daidzein-degrading bacteria was mainly based on anaerobic bacteria. Therefore, referring to previous studies, the anaerobic method was selected.
